# Design and Synthesis of Arylamidine Derivatives as Serotonin/Norepinephrine Dual Reuptake Inhibitors

**DOI:** 10.3390/molecules24030497

**Published:** 2019-01-30

**Authors:** Hui Wen, Wen Qin, Guangzhong Yang, Yanshen Guo

**Affiliations:** 1 Institute of Materia Medica, Chinese Academy of Medical Sciences & Peking Union Medical College, Beijing 100050, China; wenhui@imm.ac.cn (H.W.); chufm@imm.ac.cn (G.Y.); 2 National Institute for Nutrition and Health, Chinese Center for Disease Control and Prevention, Beijing 100050, China; qinwen@ninh.chinacdc.cn

**Keywords:** serotonin, norepinephrine, dual reuptake inhibitors, antidepressant

## Abstract

To improve the in vivo antidepressant activity of previously reported serotonin (5-HT) and norepinephrine (NE) dual reuptake inhibitors, three series of arylamidine derivatives were designed and synthesized. The in vitro 5-HT and NE reuptake inhibitory activities of these compounds were evaluated, and compound **II-5** was identified as the most potent 5-HT (IC_50_ = 620 nM) and NE (IC_50_ = 10 nM) dual reuptake inhibitor. Compound **II-5** exhibited potent antidepressant activity in the rat tail suspension test and showed an acceptable safety profile in a preliminary acute toxicity test in mice. Our results show that these arylamidine derivatives exhibit potent 5-HT/NE dual reuptake inhibition and should be explored further as antidepressant drug candidates.

## 1. Introduction

Depression is a common mental illness that can be severe, chronic, and sometimes life-threatening [1,2]. According to the World Health Organization, depression is one of the top causes of disability worldwide and affects 350 million people [3]. Therefore, there is an urgent need to develop antidepressants to improve the lives of people living with depression [4,5,6,7]. 

**Figure 1:**
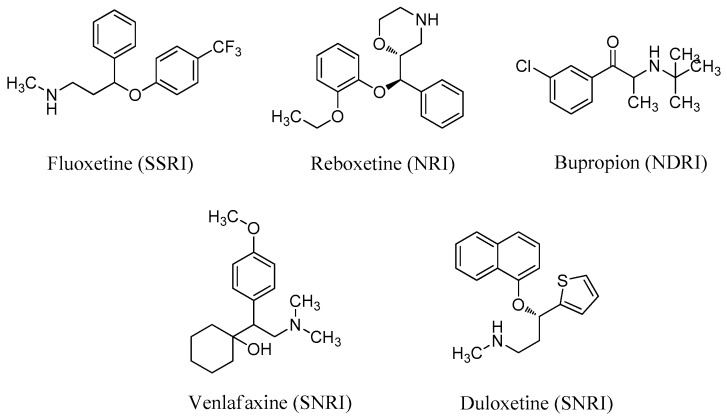
Structures of antidepressants with single or dual reuptake inhibition.

The biogenic amine transmitters, serotonin (5-HT), norepinephrine (NE), and dopamine (DA), are closely related to symptoms of depression [8]. First-generation antidepressants were mainly designed as single reuptake inhibitors, such as selective 5-HT reuptake inhibitors (SSRIs) and NE reuptake inhibitors (NRIs). Several single reuptake inhibitors, such as fluoxetine (SSRI) and reboxetine (NRI) (Figure 1), have poor safety and tolerability profiles. Second-generation antidepressants are dual reuptake inhibitors, such as 5-HT/NE reuptake inhibitors (SNRIs) and NE/DA reuptake inhibitors (NDRIs). Dual inhibitors currently on the market include bupropion (NDRI), venlafaxine (SNRI), and duloxetine (SNRI) (Figure 1). Dual reuptake inhibitors have better antidepressant effects and fewer side effects than single reuptake inhibitors [9]. SNRIs have a shorter working time and fewer adverse reactions, and thus are regarded as better antidepressant drugs than SSRIs [10].

**Figure 2:**
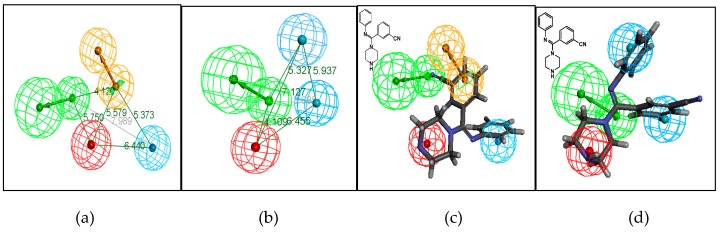
Proposed pharmacophore model Hypo1 of serotonin (5-HT) (**a**) and norepinephrine (NE) (**b**) produced by the Hypo Gen module in Catalyst 4.10 software (Accelrys, San Diego, CA, USA). The distance between the pharmacophore features is reported in angstroms. Mapping of an arylamidine derivative onto the 5-HT (**c**) and NE (**d**) pharmacophore models. Green, blue, brown, and red contours represent hydrogen-bond acceptor (HA), hydrophobic (HY), aromatic ring (RA), and positive ionizable (PI) pharmacophore features, respectively.

**Figure 3:**
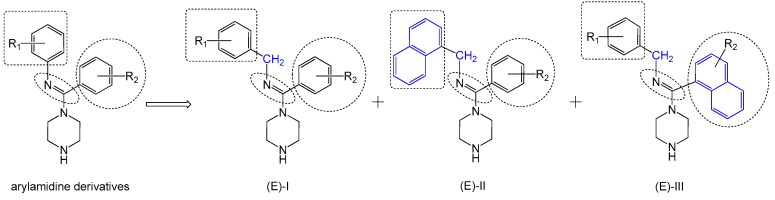
Design of inhibitor series I–III based on arylamidine derivatives.

In our previous study, we used compounds with 5-HT or NE reuptake inhibition activities to build a pharmacophore model of the characteristics of 5-HT and NE inhibitors (Figure 2) [11]. A series of substituted arylamidine derivatives was discovered using the 5-HT and NE pharmacophore model (Figure 3). One type of arylamidine derivative fitted the proposed 5-HT and NE pharmacophore model. Subsequent pharmacological tests indicated that these compounds showed good 5-HT and NE reuptake inhibition activity [12,13,14].

However, an in vivo study of antidepressant activity in rats showed that arylamidine derivatives were less potent than the commercial SNRI antidepressants venlafaxine and duloxetine. We suspected that the high rigidity of the planar structure in arylamidine derivatives, compared with the structures of other SNRI inhibitors, such as venlafaxine and duloxetine, was a main cause of the poor antidepressant activity in rats. To circumvent these problems with arylamidine derivatives, in this work, we designed and synthesized 32 compounds in three series (I–III) of optimized arylamidine derivatives. The pharmacological activity of the 32 compounds was evaluated in vitro and selected compounds were evaluated in vivo.

## 2. Results and Discussion

### 2.1. Chemical Design and Modeling Prediction

To increase the structural flexibility of the optimized compounds, we designed three series of compounds (Figure 3). A methylene group (-CH_2_-) was inserted between the aromatic ring and nitrogen atom to form the structure of series I. Then, based on series I, another two series of derivatives were designed. In series II, the benzyl ring was replaced with a naphthalene ring and in series III, the amidine ring was replaced with a naphthalene ring.

**Figure 4:**
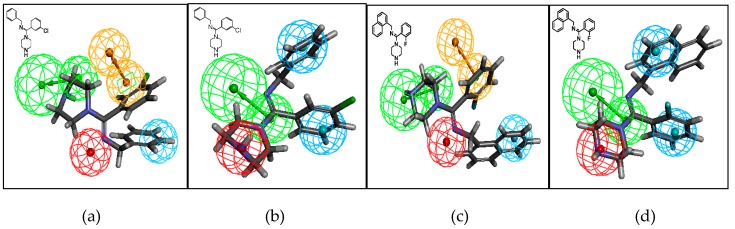
Mapping of compounds **I-4** and **II-4** on the serotonin (5-HT) pharmacophore model (**a**,**c**) and on the NE pharmacophore model (**b**,**d**). Green, blue, brown, and red contours represent hydrogen-bond acceptor (HA), hydrophobic (HY), aromatic ring (RA), and positive ionizable (PI) pharmacophore features, respectively.

The compounds were docked into the 5-HT and NE pharmacophore models, and all three types of molecules matched the model well. Compounds **I-4** and **II-4** are shown as examples of mapping on the pharmacophore model in Figure 4. Generally, the binding, as in the 5-HT and NE pharmacophore models, can be classified as aromatic ring (RA, brown), hydrophobic (HY, blue), positive ionizable (PI, red), and hydrogen-bond acceptor (HA, green) features. Compound **I-4** was docked in the 5-HT model, and the two aromatic rings matched either the RA or HY features well. The nitrogen atoms of the piperazine ring were recognized as an HA feature, and the amidine imino nitrogen atom was recognized as a PI feature. Compound **I-4** was also mapped on the NE model and the two aromatic rings matched the HY feature, the nitrogen atoms of the piperazine ring were recognized as the PI feature, and amidine imino nitrogen atom was recognized as the HA feature. We observed similar mapping for compound **II-4** in both models. 

Our experience suggested that for the design of a new series of compounds (Figure 2), the major pharmacodynamic domain in the arylamidine derivatives, including the (*E*) configuration, would have to be retained. Only small changes to the structure of arylamidine derivatives are tolerated. Otherwise, the new compounds would lose their inhibitory activities against both 5-HT and NE reuptake. Thus, in the design, the key change was the addition of a methylene group, which broke the original planar configuration and increased the flexibility of the molecules. This design strategy balanced the pharmacological activity and the desired structural flexibility. Additionally, based on the pharmacophore models, the space for the aromatic ring in the arylamidine derivatives can tolerate a bigger aromatic fragment, such as an aphthalene ring. This might further favor the binding of the new compounds to either 5-HT or NE.

In summary, the docking study showed that all of the designed compounds matched the 5-HT and NE pharmacophore models well. The fragments in the compounds matched the corresponding binding domains in both models. Similar binding modes were observed for the mapping of compounds **I-4** and **II-4** in both models. The docking results indicated that the designed compounds were likely to be good dual reuptake inhibitors of 5-HT and NE. 

### 2.2. Chemistry Synthesis

**Scheme 1:**
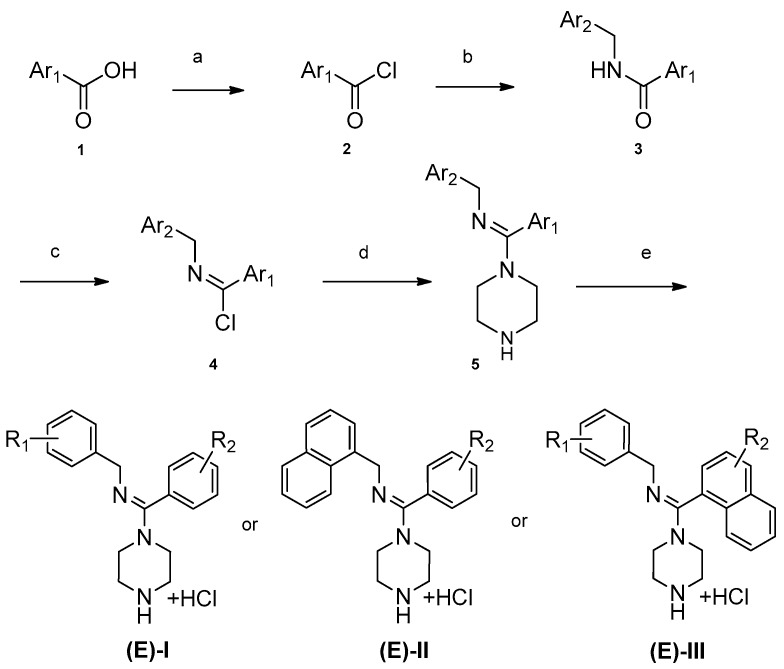
Synthesis of **(*E*)-I**–**(*E*)-III**. (a) Oxalyl chloride, CH_2_Cl_2_; (b) Ar_2_-methylamine, triethylamine, CH_2_Cl_2_; (c) PCl_5_, C_2_H_4_Cl_2_; (d) piperazine, CH_2_Cl_2_; (e) ethanol, HCl.

The synthesis for series I–III is shown in Scheme 1. An aromatic acid (**1**) was used as the starting reagent to form an acyl chloride (**2**) with yields of 75–95%. The intermediate (**2**) was reacted with various aromatic methylamines to form a substituted amide (**3**). The most important step in the scheme was the synthesis of compound **4**. All the final compounds had an (*E*) configuration, which was initially formed in the production of acyl chloride **4**. (*E*)-acyl chloride **4** was obtained by the reaction between amide **3** and PCl_5_. During the synthesis of compound **5**, piperazine could react with two molecules of compound **4** to produce disubstituted by-products. The amount of disubstituted by-products was reduced by increasing the amount of piperazine in this reaction. Subsequently, all target compounds were prepared as hydrochloride salts **(*E*)-I**, **(*E*)-II**, and **(*E*)-III** to obtain stable solids.

### 2.3. Structure Confirmation

**Figure 5:**
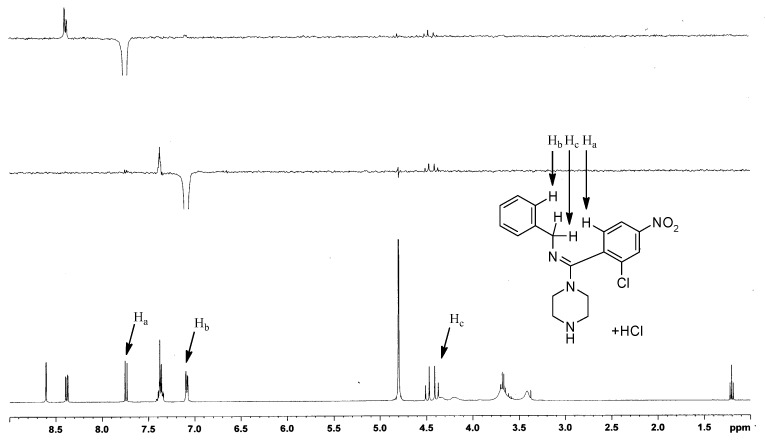
1D-NOE NMR of compound **(*E*)-I-11**.

1D-NOE NMR was used to confirm the configuration of the structure. The 1D-NOE spectrum of compound **I-11** (Figure 5) showed weak NOE enhancements between the H_a_ and H_b_ protons, and H_a_ and H_c_ protons, indicating that the H_a_ proton was spatially close to the H_b_ and H_c_ protons. Thus, the absolute configuration of the target compound was (*E*).

**Figure 6:**
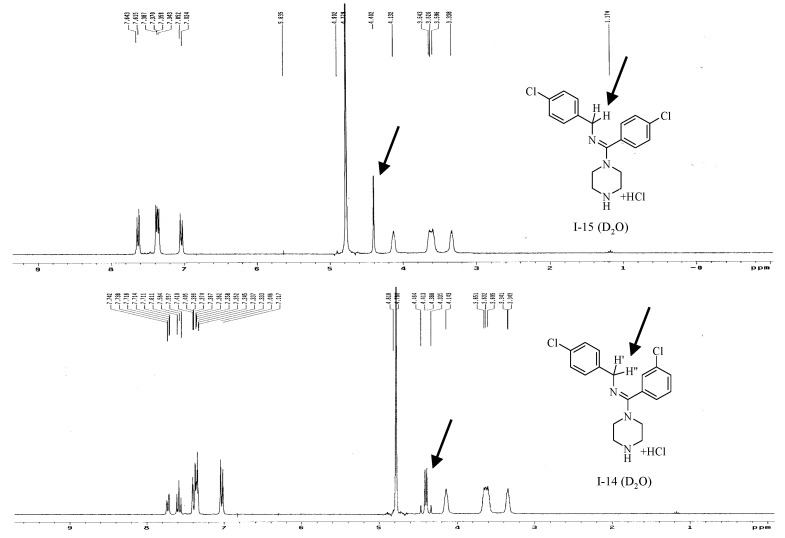
^1^H-NMR spectra of **I-14** and **I-15**.

In the ^1^H-NMR spectra of several compounds, such as **I-15**, the Ar-CH_2_-N fragment exhibited a singlet, where as in the spectra of other compounds, such as **I-14**, the same fragment produced a double doublet. The only structural difference between **I-14** and **I-15** was the *ortho*- or *para*-chloro substituent on the amidine ring (Figure 6). The difference between the spectra may arise from the weak steric hindrance caused by the *ortho*-chloro substituent in **I-14**, but not by the *para*-chloro substituent in **I-15**. The steric hindrance would make the two protons in the Ar-CH_2_-N fragment magnetically inequivalent in **I-14**, producing the double doublet. This observation is also important because if the configuration of the final compound was (*Z*), and there would be no steric hindrance, the two Ar-CH_2_-N protons would not be magnetically inequivalent, and the double doublet would not appear. Consequently, the steric hindrance indirectly indicated that the absolute configuration of the final compound was (*E*).

### 2.4. Biological Results and Discussion

#### 2.4.1. In Vitro Test

All the target compounds were evaluated for their inhibitory activity against the reuptake of 5-HT and NE at the cellular level. Rat brain tissue was used for the uptake test, which included ^3^H-5-HT and ^3^H-NE [15]. 1-[7,8-^3^*H*]-Noradrenaline and 5-hydroxy-[^3^*H*]-tryptamine trifluoroacetate were obtained from Amersham Corporation. Duloxetine was used as the reference compound. All compounds were tested at a concentration of 10 μM, and potent compounds (inhibition >80%) were assayed to obtain IC_50_ values (Table 1).

In series I, most compounds exhibited moderate dual reuptake inhibition activity. Compound **I-19** was the most potent dual inhibitor in this series (5-HT, IC_50_ = 830 nM; NE, IC_50_ = 560 nM), and was comparable to the reference compound, duloxetine (5-HT, IC_50_ = 640 nM; NE, IC_50_ = 49 nM). In series II, compounds **II-4** (5-HT, IC_50_ = 130 nM; NE, IC_50_ = 880 nM) and **II-5** (5-HT, IC_50_ = 620 nM; NE, IC_50_ = 10 nM) were the most potent compounds. Most compounds in this series had improved dual reuptake inhibition activity. In series III, compound **III-1** also showed good potency for 5-HT and NE transporters.

The series I compounds maintained the dual 5-HT and NE inhibitory activity after the insertion of a methylene into the arylamidine derivative structure, although the general inhibitory activity was moderate. Various substituents on the benzene ring did not dramatically improve the binding of these compounds with the targets. In series II, a naphthalene ring replaced the benzyl ring. Interestingly, some compounds in this series exhibited dramatically improved dual inhibitory activities. Thus, the addition of a big hydrophobic fragment, that is, the naphthalene ring, improved binding to the5-HT and NE transporters. 

#### 2.4.2. In Vivo Test

**Figure 7:**
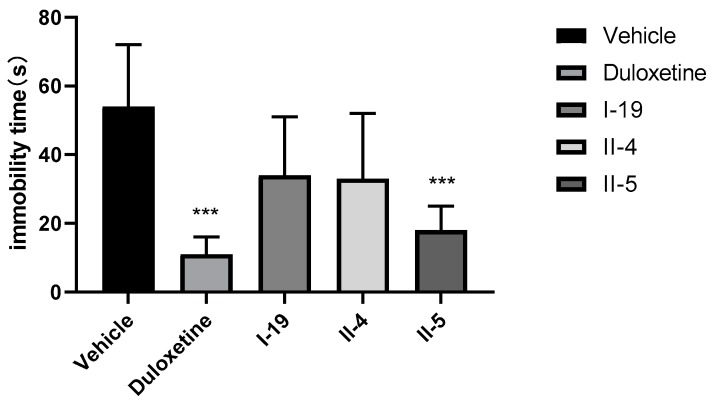
Tail suspension test (TST) results of compounds **I-19**, **II-4**, and **II-5** and duloxetine (single dose of 30 mg/kg), (*** *p* < 0.001).

Based on the in vitro results, compounds **I-19**, **II-4**, and **II-5** were selected for the tail suspension test (TST) in rats. The TST has been used widely as a preclinical model for screening antidepressant activity, and it is sensitive to commercially available antidepressant drugs. In the assay, rats are suspended by tail and the time that the animal is immobile is recorded. The compounds were administered to male rats at a dose of 30 mg/kg (PO). Duloxetine was used as the positive control (30 mg/kg, PO). The compounds all reduced the immobility times of rats compared with the negative control vehicle group, indicating that all compounds exerted an antidepressant effect (Figure 7, Table 2). However, the antidepressant effect of compound **II-5** was the closest to that of duloxetine, although it was still smaller. Both compound **II-5** and duloxetine exhibited statistical significance for the antidepressant effect. In addition, the safety profile of compound **II-5** was also explored with a preliminary acute toxicity test in mice at a single dose of 200 or 400 mg/kg (PO) (*n* = 6). Compound **II-5** did not result in the death of mice at doses of 200 and 400 mg/kg after 20 h, and thus the LD_50_ was >400 mg/kg (PO), indicating that the toxicity was low and that the safety profile was acceptable (Table 3). 

In summary, compound **II-5** had the most potent in vitro 5-HT and NE dual inhibitory activity of the three compounds used in the TST, and it also had the most potent in vivo pharmacological antidepressant effect. Compound **II-5** was reasonably safe for mice at a dose of 30 mg/kg. However, the in vivo antidepressant effect of compound **II-5** (5-HT, IC_50_ = 620 nM; NE, IC_50_ = 10 nM) was smaller than that of leading antidepressant duloxetine (5-HT, IC_50_ = 640 nM; NE, IC_50_ = 49 nM), although they had similar in vitro activities. The less potent in vivo antidepressant effect of compound **II-5** may arise from its in vivo metabolism, which needs to be examined and optimized. 

## 3. Experiment Section

### 3.1. Chemistry

Most reagents and solvents were obtained from commercial suppliers and used as received. Some reactions were performed under an inert atmosphere (N_2_). ^1^H-NMR spectra were obtained on an NMR spectrometer (Mercury, Varian, San Diego, CA, USA; 400MHz). Melting points were measured with a melting point apparatus (Yanaco-50, YANACO company, Kyoto, Japan) and are not corrected. Electro spray ionization (ESI) mass spectra and high-resolution mass spectroscopy (HRMS) were performed with a liquid chromatograph/mass selective detector time-of-flight mass spectrometer (LC/MSD TOF, Agilent Technologies, Santa Clara, CA, USA). The yields are of the purified product and are not optimized. Silica gel column chromatography was performed with silica gel 60G (Qingdao Haiyang Chemical, Qingdao, China). The procedure for preparing series I–III was the same as for compound **I-1**. Purity was determined using HPLC, LC/MS and NMR spectroscopy. All of the synthesized compounds have purity over 95%.

#### General Procedure for Preparing (*E*)-*N*-((4-Methoxyphenyl)(piperazin-1-yl)methylene)-1-phenylmethanamine hydrochloride (**I-1**)

##### Preparation of *N*-Benzyl-4-methoxybenzamide

One drop of dry *N*,*N*-dimethyl formamide and oxalyl chloride (0.62 g, 4.9 mmol) were added dropwise to a solution of 4-methoxybenzoic acid (0.50 g, 3.3 mmol) in dry CH_2_Cl_2_ (20 mL). The reaction mixture was stirred at room temperature for 2 h, and then distilled in vacuo to give the crude acyl chloride, which was dissolved in dry CH_2_Cl_2_ (10 mL). Dry triethylamine (0.40 g, 3.9 mmol) was added to a solution of benzylamine (0.35 g, 3.3 mmol) in dry CH_2_Cl_2_ (20 mL). The reaction mixture was cooled to 0 °C, and the acyl chloride solution was added dropwise. After stirring at 20–25 °C for 4 h, the reaction mixture was distilled in vacuo, and washed with a 10% solution of NaOH and water, and dried. Silica gel column chromatography (hexane/ethyl acetate) afforded **I-1** as a white powder (0.75 g, 94.8% yield).

##### Preparation of (*E*)-*N*-((4-Methoxyphenyl)(piperazin-1-yl)methylene)-1-phenylmethanamine hydrochloride (**I-1**)

*N*-Benzyl-4-methoxybenzamide (0.50 g, 2.1 mmol) was dissolved in dry dichloroethane (10 mL), and the solution was heated to 50 °C for 30 min. PCl_5_ (0.44 g, 2.1 mmol) was added in one portion and the reaction was stirred at 90 °C for 2 h. After most of the solvent was removed in vacuo, the residue was dissolved in dry CH_2_Cl_2_ (10 mL) and was added dropwise to a solution of piperazine (0.54 g, 6 mmol) in dry CH_2_Cl_2_ (10 mL) at 0 °C. The reaction mixture was stirred at room temperature for 2 h, and then distilled in vacuo. Et_2_O (50 mL) and saturated aqueous Na_2_CO_3_ (30 mL) were added to the mixture. After the aqueous extract was separated, the organic extract was washed twice with saturated aqueous Na_2_CO_3_ and 10% aqueous NaOH, and then extracted with 1 mol/L HCl. The aqueous extract was washed twice with CH_2_Cl_2_ and Et_2_O, and was treated with 10% aqueous NaOH to increase the pH to 10. The aqueous extract was re-extracted with Et_2_O (50 mL), and the Et_2_O extract was dried over Na_2_SO_4_. After Et_2_O was removed under vacuum, the residue was dissolved in dry ethanol (5 mL), and an ethanolic solution of HCl was added dropwise to adjust the pH to 2 to afford **I-1** as a white powder (0.18 g, 25% yield). Mp: 195–197 °C. ^1^H-NMR (400 MHz, D_2_O): δ 3.37 (br, 2H, piperazinyl-H), 3.61 (br, 2H, piperazinyl-H), 3.66 (br, 2H,piperazinyl-H), 3.89 (s, 3H, -CH_3_), 4.13 (br, 2H, piperazinyl-H), 4.44 (s, 2H, -CH_2_-), 7.10–7.16 (m, 4H, ArH), 7.35–7.41 (m, 5H, ArH). HRMS calcd for C_19_H_23_N_3_O (M + H)^+^, 310.1914; found, 310.1912.

*(E)-N-((4-Nitrophenyl)(piperazin-1-yl)methylene)-1-phenylmethanaminehydrochloride (***I-2***)*: Yield 40%, Mp: 230–231 °C. ^1^H-NMR (400 MHz, D_2_O): δ 3.37 (br, 2H, piperazinyl-H), 3.61 (br, 2H, piperazinyl-H), 3.66 (br, 2H, piperazinyl-H), 4.13 (br, 2H, piperazinyl-H), 4.44 (s, 2H, -CH_2_-), 7.10–7.16 (m, 4H, ArH), 7.35–7.41 (m, 5H, ArH). HRMS calcd for C_18_H_20_N_4_O_2_ (M + H)^+^, 325.1659; found, 325.1654.

*(E)-N-((2-Chlorophenyl)(piperazin-1-yl)methylene)-1-phenylmethanaminehydrochloride (***I-3***)*: Yield 18%, Mp: 142–144 °C.^1^H-NMR (400 MHz, D_2_O): δ 3.40 (br, 2H, piperazinyl-H), 3.67 (br, 4H, piperazinyl-H), 4.24 (br, 2H, piperazinyl-H), 4.38 (d, 1H, *J* = 14.0 Hz, -CH′-), 4.46 (d, 1H, *J* = 14.0 Hz, -CH′′-), 7.11–7.12 (m, 2H, ArH), 7.37–7.38 (m, 2H, ArH), 7.48 (d, 1H, *J* = 7.6 Hz, ArH), 7.55–7.58 (m, 2H, ArH), 7.70–7.73 (m, 2H, ArH). HRMS calcd for C_18_H_20_ClN_3_ (M + H)^+^, 314.1419; found, 314.1421. 

*(E)-N-((3-Chlorophenyl)(piperazin-1-yl)methylene)-1-phenylmethanaminehydrochloride (***I-4***)*: Yield 66%, Mp: 235–237 °C. ^1^H-NMR (400 MHz, D_2_O): δ 3.34 (t, 2H, *J* = 5.4 Hz, piperazinyl-H), 3.58–3.66 (m, 4H, piperazinyl-H), 4.14 (t, 2H, *J* = 5.4 Hz, piperazinyl-H), 4.39 (d, 1H, *J* = 15.9 Hz, -CH′-), 4.45 (d, 1H, *J* = 15.9 Hz, -CH′′-), 7.07–7.11 (m, 2H, ArH), 7.35–7.38 (m, 4H, ArH), 7.45 (s, 1H, ArH), 7.56–7.61 (m, 1H, ArH), 7.73 (d, 1H, *J* = 7.2 Hz, ArH). HRMS calcd for C_18_H_20_ClN_3_ (M + H)^+^, 314.1419; found, 314.1411.

*(E)-N-((4-Chlorophenyl)(piperazin-1-yl)methylene)-1-phenylmethanaminehydrochloride (***I-5***)*: Yield 65%, Mp: 182–184 °C. ^1^H- NMR (400 MHz, D_2_O): δ 3.35 (br, 2H, piperazinyl-H), 3.64 (br, 4H, piperazinyl-H), 4.14 (br, 2H, piperazinyl-H), 4.42 (s, 2H, -CH_2_-), 7.11 (br, 2H, ArH), 7.35–7.42 (m, 5H, ArH), 7.63 (d, 2H, *J* = 8.4 Hz, ArH). HRMS calcd for C_18_H_20_ClN_3_ (M + H)^+^, 314.1419; found, 314.1414.

*(E)-N-((4-Fluorophenyl)(piperazin-1-yl)methylene)-1-phenylmethanaminehydrochloride (***I-6***)*: Yield 33%, Mp: 220–222 °C. ^1^H-NMR (400 MHz, D_2_O): δ 3.34 (br, 2H, piperazinyl-H), 3.58 (br, 2H, piperazinyl-H), 3.64 (br, 2H, piperazinyl-H), 4.12 (br, 2H, piperazinyl-H), 4.43 (s, 2H, -CH_2_-), 7.11 (br, 2H, ArH), 7.32–7.37 (m, 5H, ArH), 7.46–7.50 (m, 2H, ArH). HRMS calcd for C_18_H_20_FN_3_ (M + H)^+^, 298.1714; found, 298.1710.

*(E)-N-((2-Fluorophenyl)(piperazin-1-yl)methylene)-1-phenylmethanaminehydrochloride (***I-7***)*: Yield 22%, Mp: 182–184 °C. ^1^H-NMR (400 MHz, D_2_O): δ 3.34 (br, 2H, piperazinyl-H), 3.59 (br, 2H, piperazinyl-H), 3.71 (br, 2H, piperazinyl-H), 4.17 (br, 2H, piperazinyl-H), 4.42 (d, 1H, *J* = 15.6 Hz, -CH′′-), 4.49 (d, 1H, *J* = 15.6 Hz, -CH′′-), 7.08–7.10 (m, 2H, ArH), 7.34–7.39 (m, 4H, ArH), 7.43–7.45 (m, 2H, ArH), 7.72–7.80 (m, 1H, ArH). HRMS calcd for C_18_H_20_FN_3_ (M + H)^+^, 298.1714; found, 298.1706.

*(E)-4-((Benzylimino)(piperazin-1-yl)methyl)benzonitrilehydrochloride (***I-8***)*: Yield 38%, Mp: 205–207 °C. ^1^H-NMR (400 MHz, D_2_O): δ 3.36 (br, 2H, piperazinyl-H), 3.61 (br, 4H, piperazinyl-H), 4.17 (br, 2H, piperazinyl-H), 4.40 (s, 2H, -CH_2_-), 7.04–7.08 (m, 2H, ArH), 7.34–7.36 (m, 3H, ArH), 7.60 (d, 2H, *J* = 8.4 Hz, ArH), 7.98 (d, 2H, *J* = 8.4 Hz, ArH). HRMS calcd for C_19_H_20_N_4_ (M + H)^+^, 305.1761; found, 305.1760.

*(E)-3-((Benzylimino)(piperazin-1-yl)methyl)benzonitrilehydrochloride (***I-9***)*: Yield 60%, Mp: 190–192 °C. ^1^H-NMR (400 MHz, D_2_O): δ 3.35 (br, 2H, piperazinyl-H), 3.61 (br, 4H, piperazinyl-H), 4.18 (br, 2H, piperazinyl-H), 4.36 (d, 1H, *J* = 15.6 Hz, -CH′-), 4.44 (d, 1H, *J* = 15.6 Hz, -CH′′-), 7.04–7.06 (m, 2H, ArH), 7.31–7.41 (m, 3H, ArH), 7.72–7.81 (m, 3H, ArH), 8.07 (d, 1H, *J* = 6.9 Hz, ArH). HRMS calcd for C_19_H_20_N_4_ (M + H)^+^, 305.1761; found, 305.1758.

*(E)-1-Phenyl-N-(piperazin-1-yl(p-tolyl)methylene)methanaminehydrochloride (***I-10***)*: Yield 50%, Mp: 172–174 °C. ^1^H-NMR (400 MHz, D_2_O): δ 2.42 (s, 3H, -CH_3_), 3.34 (t, 2H, *J* = 5.4 Hz, *J* = 5.1 Hz, piperazinyl-H), 3.59 (t, 2H, *J* = 5.7 Hz, *J* = 5.1 Hz, piperazinyl-H), 3.64 (t, 2H, *J* = 5.7 Hz, piperazinyl-H), 4.12 (t, 2H, *J* = 6.0 Hz, piperazinyl-H), 4.42 (s, 2H, -CH_2_-), 7.09–7.12 (m, 2H, ArH), 7.30–7.37 (m, 5H, ArH), 7.45 (d, 2H, *J* = 8.1 Hz, ArH). HRMS calcd for C_19_H_23_N_3_ (M + H)^+^, 294.1965; found, 294.1962.

*(E)-N-((2-Chloro-4-nitrophenyl)(piperazin-1-yl)methylene)-1-phenylmethanaminehydrochloride (***I-11***)*: Yield 73%, Mp: 195–197 °C. ^1^H-NMR (400 MHz, D_2_O): δ 3.39 (br, 2H, piperazinyl-H), 3.66 (br, 4H, piperazinyl-H), 4.13 (br, 2H, piperazinyl-H), 4.35 (d, 1H, *J* = 15.6 Hz, -CH′-), 4.46 (d, 1H, *J* = 15.6 Hz, -CH′′-), 7.05 (d, 2H, *J* = 7.8 Hz, ArH), 7.31–7.38 (m, 3H, ArH), 7.71 (d, 1H, *J* = 8.7 Hz, ArH), 8.35 (d, 1H, *J* = 8.4 Hz, ArH), 8.57 (s, 1H, *J* = 8.7 Hz, ArH). HRMS calcd for C_18_H_19_ClN_4_O_2_ (M + H)^+^, 359.1269; found, 359.1277.

*(E)-1-(4-Chlorophenyl)-N-((4-nitrophenyl)(piperazin-1-yl)methylene)methanaminehydrochloride **(*****I-12***)*: Yield 22%, Mp: 172–174 °C. ^1^H-NMR (400 MHz, D_2_O): δ 3.35 (br, 2H, piperazinyl-H), 3.65 (br, 4H, piperazinyl-H), 4.18 (br, 2H, piperazinyl-H), 4.39 (s, 2H, -CH_2_-), 7.01 (d, 2H, *J* = 8.4 Hz, ArH), 7.34 (d, 2H, *J* = 8.4 Hz, ArH), 7.67 (d, 2H, *J* = 8.7 Hz, ArH), 8.42 (d, 2H, *J* = 8.7 Hz, ArH). HRMS calcd for C_18_H_19_ClN_4_O_2_ (M + H)^+^, 359.1269; found, 359.1271.

*(E)-1-(4-Chlorophenyl)-N-((2-chlorophenyl)(piperazin-1-yl)methylene)methanaminehydrochloride (***I-13***)*: Yield 56%, Mp: 181–183 °C. ^1^H-NMR (400 MHz, D_2_O): δ 3.36 (br, 2H, piperazinyl-H), 3.61 (br, 4H, piperazinyl-H), 4.14 (br, 2H, piperazinyl-H), 4.34 (d, 1H, *J* = 15.6 Hz, -CH′-), 4.42 (d, 1H, *J* = 15.6 Hz, -CH′′-), 7.01 (d, 2H, *J* = 8.4 Hz, ArH), 7.33 (d, 2H, *J* = 8.4 Hz, ArH), 7.42 (d, 1H, *J* = 7.8 Hz, ArH), 7.55 (t, 1H, *J* = 7.5 Hz, ArH), 7.64–7.72 (m, 2H, ArH). HRMS calcd for C_18_H_19_Cl_2_N_3_ (M + H)^+^, 348.1029; found, 348.1032.

*(E)-1-(4-Chlorophenyl)-N-((3-chlorophenyl)(piperazin-1-yl)methylene)methanaminehydrochloride (***I-14***)*: Yield 53%, Mp: 218–220 °C. ^1^H-NMR (400 MHz, D_2_O): δ 3.34 (br, 2H, piperazinyl-H), 3.63 (br, 4H, piperazinyl-H), 4.14 (br, 2H, piperazinyl-H), 4.35 (d, 1H, *J* = 15.9 Hz, -CH′-), 4.35 (d, 1H, *J* = 15.9 Hz, -CH′′-), 7.03 (d, 2H, *J* = 8.7 Hz, ArH), 7.33–7.37 (m, 3H, ArH), 7.40 (s, 1H, ArH), 7.55–7.61 (m, 1H, ArH), 7.72 (d, 1H, *J* = 7.2 Hz, ArH). HRMS calcd for C_18_H_19_Cl_2_N_3_ (M + H)^+^, 348.1029; found, 348.1027.

*(E)-1-(4-Chlorophenyl)-N-((4-chlorophenyl)(piperazin-1-yl)methylene)methanaminehydrochloride (***I-15***)*: Yield 36%, Mp: 157–159 °C. ^1^H-NMR (400 MHz, D_2_O): δ 3.33 (br, 2H, piperazinyl-H), 3.62 (br,4H, piperazinyl-H), 4.13 (br, 2H, piperazinyl-H), 4.40 (s, 2H, -CH_2_-), 7.03 (d, 2H, *J* = 8.4 Hz, ArH), 7.35 (d, 2H, *J* = 8.1 Hz, ArH), 7.37 (d, 2H, *J* = 8.1 Hz, ArH), 7.63 (d, 2H, *J* = 8.4 Hz, ArH). HRMS calcd for C_18_H_19_Cl_2_N_3_ (M + H)^+^, 348.1029; found, 348.1027.

*(E)-1-(4-Chlorophenyl)-N-((4-fluorophenyl)(piperazin-1-yl)methylene)methanaminehydrochloride (***I-16***)*: Yield 20%, Mp: 165–167 °C. ^1^H-NMR (400 MHz, D_2_O): δ 3.34 (br, 2H, piperazinyl-H), 3.62 (br, 4H, piperazinyl-H), 4.13 (br, 2H, piperazinyl-H), 4.41 (s, 2H, -CH_2_-), 7.05 (d, 2H, *J* = 8.1 Hz, ArH), 7.31–7.37 (m, 4H, ArH), 7.42–7.47 (m, 2H, ArH). HRMS calcd for C_18_H_19_ClFN_3_ (M + H)^+^, 332.1324; found, 332.1323.

*(E)-1-(4-Chlorophenyl)-N-((2-fluorophenyl)(piperazin-1-yl)methylene)methanaminehydrochloride (***I-17***)*: Yield 21%, Mp: 215–217 °C. ^1^H-NMR (400 MHz, D_2_O): δ 3.26 (br, 2H, piperazinyl-H), 3.52 (br, 2H, piperazinyl-H), 3.70 (br, 2H, piperazinyl-H), 4.18 (br, 2H, piperazinyl-H), 4.41 (d, 1H, *J* = 15.6 Hz, -CH′-), 4.47 (d, 1H, *J* = 15.6 Hz, -CH′′-), 7.02 (d, 2H, *J* = 8.4 Hz, ArH), 7.32–7.38 (m, 3H, ArH), 7.41–7.43 (m, 2H, ArH), 7.72–7.79 (m, 1H, ArH). HRMS calcd for C_18_H_19_ClFN_3_ (M + H)^+^, 332.1324; found, 332.1321.

*(E)-1-(4-Chlorophenyl)-N-((4-cyanophenyl)(piperazin-1-yl)methylene)methanaminehydrochloride (***I-18***)*: Yield 39%, Mp: 180–182 °C. ^1^H-NMR (400 MHz, D_2_O): δ 3.35 (br, 2H, piperazinyl-H), 3.62 (br, 4H, piperazinyl-H), 4.16 (br, 2H, piperazinyl-H), 4.37 (s, 2H, -CH_2_-), 7.01 (d, 2H, *J* = 8.4 Hz, ArH), 7.34 (d, 2H, *J* = 8.4 Hz, ArH), 7.58 (d, 2H, *J* = 8.7 Hz, ArH), 7.97 (d, 2H, *J* = 8.7 Hz, ArH). HRMS calcd for C_19_H_19_ClN_4_ (M + H)^+^, 339.1371; found, 339.1368.

*(E)-1-(4-Chlorophenyl)-N-((3-cyanophenyl)(piperazin-1-yl)methylene)methanaminehydrochloride (***I-19***)*: Yield 26%, Mp: 156–158 °C. ^1^H-NMR (400 MHz, D_2_O): δ 3.21 (br, 2H, piperazinyl-H), 3.49 (br,4H, piperazinyl-H), 4.03 (br, 2H, piperazinyl-H), 4.21 (d, 1H, *J* = 15.9 Hz, -CH′-), 4.29 (d, 1H, *J* = 15.9 Hz, -CH′′-), 6.85 (d, 2H, *J* = 8.7 Hz, ArH), 7.21 (d, 2H, *J* = 8.7 Hz, ArH), 7.56–7.67 (m, 3H, ArH), 7.93 (d, 1H, *J* = 7.8 Hz, ArH). HRMS calcd for C_19_H_19_ClN_4_ (M + H)^+^, 339.1371; found, 339.1366.

*(E)-1-(4-Chlorophenyl)-N-(piperazin-1-yl(p-tolyl)methylene)methanaminehydrochloride (***I-20***)*: Yield 57%, Mp: 183–185 °C. ^1^H-NMR (400 MHz, D_2_O): δ 2.41 (s, 3H, -CH_3_), 3.32 (br, 2H, piperazinyl-H), 3.58 (br, 2H, piperazinyl-H), 3.63 (br, 2H, piperazinyl-H), 4.11 (br, 2H, piperazinyl-H), 4.40 (s, 2H, -CH_2_-), 7.04 (d, 2H, *J* = 8.1 Hz, ArH), 7.27 (d, 2H, *J* = 8.4 Hz, ArH), 7.35 (d, 2H, *J* = 8.4 Hz, ArH), 7.43 (d, 2H, *J* = 8.1 Hz, ArH). HRMS calcd for C_19_H_22_ClN_3_ (M + H)^+^, 328.1575; found, 328.1574.

*(E)-1-(4-Methoxyphenyl)-N-((4-nitrophenyl)(piperazin-1-yl)methylene)methanaminehydrochloride (***I-21***)*: Yield 15%, Mp: 210–212 °C. ^1^H-NMR (400 MHz, D_2_O): δ 3.33 (br, 2H, piperazinyl-H), 3.59 (br, 4H, piperazinyl-H), 3.81 (s, 3H, -OCH_3_), 4.15 (br, 2H, piperazinyl-H), 4.35 (s, 2H, -CH_2_-), 6.90 (d, 2H, *J* = 8.7 Hz, ArH), 6.97 (d, 2H, *J* = 8.7 Hz, ArH), 7.66 (d, 2H, *J* = 9.3 Hz, ArH), 8.42 (d, 2H, *J* = 9.3 Hz, ArH). HRMS calcd for C_19_H_22_N_4_O_3_ (M + H)^+^, 355.1765; found, 355.1766.

*(E)-1-(Naphthalen-1-yl)-N-(phenyl(piperazin-1-yl)methylene)methanaminehydrochloride (***II-1***)*: Yield 34%, Mp: 187–189 °C. ^1^H-NMR (400 MHz, D_2_O): δ 3.33 (t, 2H, *J* = 5.4 Hz, piperazinyl-H), 3.54–3.68 (m, 4H, piperazinyl-H), 4.12 (t, 2H, *J* = 5.4 Hz, piperazinyl-H), 4.82 (s, 2H, -CH_2_-), 7.22 (d, 1H, *J* = 7.2 Hz, ArH), 7.40–7.44 (m, 3H, ArH), 7.51–7.57 (m, 4H, ArH), 7.60–7.68 (m, 2H, ArH), 7.87 (d, 1H, *J* = 8.1 Hz, ArH), 7.87 (d, 1H, *J* = 9.0 Hz, ArH). HRMS calcd for C_22_H_23_N_3_ (M + H)^+^, 330.1965; found, 330.1962.

*(E)-1-(Naphthalen-1-yl)-N-((4-nitrophenyl)(piperazin-1-yl)methylene)methanaminehydrochloride (***II-2***)*: Yield 11%, Mp: 202–204 °C. ^1^H-NMR (400 MHz, D_2_O): δ 3.34 (br, 2H, piperazinyl-H), 3.60 (br, 4H, piperazinyl-H), 4.21 (br, 2H, piperazinyl-H), 4.84 (s, 2H, -CH_2_-), 7.20 (d, 1H, *J* = 6.9 Hz, ArH), 7.38–7.43 (m, 1H, ArH), 7.46–7.53 (m, 2H, ArH), 7.56–7.61 (m, 3H, ArH), 7.84–7.89 (m, 2H, ArH), 8.17 (d, 2H, *J* = 9.0 Hz, ArH). HRMS calcd for C_22_H_22_N_4_O_2_ (M + H)^+^, 375.1816; found, 375.1814.

*(E)-N-((4-Fluorophenyl)(piperazin-1-yl)methylene)-1-(naphthalen-1-yl)methanaminehydrochloride (***II-3***)*: Yield 24%, Mp: 193–195 °C. ^1^H-NMR (400 MHz, D_2_O): δ 3.36 (br, 2H, piperazinyl-H), 3.58 (br,2H, piperazinyl-H), 3.65 (t, 2H, *J* = 4.2 Hz, *J* = 5.7 Hz, piperazinyl-H), 4.12 (t, 2H, *J* = 5.1 Hz, *J* = 5.4 Hz, piperazinyl-H), 4.87 (s, 2H, -CH_2_-), 7.22–7.28 (m, 3H, ArH), 7.41–7.48 (m, 3H, ArH), 7.52–7.60 (m, 2H, ArH), 7.65–7.68 (m, 1H, ArH), 7.91 (d, 1H, *J* = 8.4 Hz, ArH), 7.95–7.98 (m, 1H, ArH). HRMS calcd for C_22_H_22_FN_3_ (M + H)^+^, 348.1871; found, 348.1873.

*(E)-N-((2-Fluorophenyl)(piperazin-1-yl)methylene)-1-(naphthalen-1-yl)methanaminehydrochloride (***II-4***)*: Yield 41%, Mp: 148–150 °C. ^1^H-NMR (400 MHz, D_2_O): δ 3.33 (br, 2H, piperazinyl-H), 3.56 (br,2H, piperazinyl-H), 3.67 (br, 2H, piperazinyl-H), 4.12–4.21 (m, 2H, piperazinyl-H), 4.92 (s, 2H, -CH_2_-), 7.12 (br, 1H, ArH), 7.23–7.29 (m, 1H, ArH), 7.33–7.46 (m, 3H, ArH), 7.56 (br, 2H, ArH), 7.64–7.72 (m, 2H, ArH), 7.88–8.05 (m, 2H, ArH). HRMS calcd for C_22_H_22_FN_3_ (M + H)^+^, 348.1871; found, 348.1868.

*(E)-N-((2-Chlorophenyl)(piperazin-1-yl)methylene)-1-(naphthalen-1-yl)methanaminehydrochloride (***II-5***)*: Yield 58%, Mp: 165–167 °C. ^1^H-NMR (400 MHz, D_2_O): δ 3.34 (br, 2H, piperazinyl-H), 3.63 (br, 4H, piperazinyl-H), 4.15 (br, 2H, piperazinyl-H), 4.89 (s, 2H, -CH_2_-), 7.07 (d, 1H, *J* = 6.6 Hz, ArH), 7.37–7.49 (m, 3H, ArH), 7.55–7.65 (m, 4H, ArH), 7.78–7.81 (m, 1H, ArH), 7.91 (d, 1H, *J* = 8.7 Hz, ArH), 7.98–8.04 (m, 1H, ArH). HRMS calcd for C_22_H_22_ClN_3_ (M + H)^+^, 364.1575; found, 364.1574.

*(E)-N-((3-Chlorophenyl)(piperazin-1-yl)methylene)-1-(naphthalen-1-yl)methanaminehydrochloride (***II-6***)*: Yield 34%, Mp: 198–200 °C. ^1^H-NMR (400 MHz, D_2_O): δ 3.31 (br, 2H, piperazinyl-H), 3.65 (br,4H, piperazinyl-H), 4.12 (br, 2H, piperazinyl-H), 4.78 (s, 2H, -CH_2_-),7.16 (d, 1H, *J* = 6.9 Hz, ArH), 7.29–7.32 (m, 2H, ArH), 7.39–7.62 (m, 6H, ArH), 7.87 (d, 1H, *J* = 8.4 Hz, ArH), 7.93 (d, 1H, *J* = 6.9 Hz, ArH). HRMS calcd for C_22_H_22_ClN_3_ (M + H)^+^, 364.1575; found, 364.1576.

*(E)-N-((4-Chlorophenyl)(piperazin-1-yl)methylene)-1-(naphthalen-1-yl)methanaminehydrochloride (***II-7***)*: Yield 41%, Mp: 210–212 °C. ^1^H-NMR (400 MHz, D_2_O): δ 3.19 (t, 2H, *J* = 5.1 Hz, *J* = 5.1 Hz, piperazinyl-H), 3.43 (t, 2H, *J* = 6.9 Hz, *J* = 3.9 Hz, piperazinyl-H), 3.50 (t, 2H, *J* = 5.7 Hz, piperazinyl-H), 4.12 (t, 2H, *J* = 6.0 Hz, piperazinyl-H), 4.75 (s, 2H, -CH_2_-), 7.09 (d, 1H, *J* = 6.6 Hz, ArH), 7.20 (d, 2H, *J* = 8.4 Hz, ArH), 7.30–7.35 (m, 1H, ArH), 7.38 (d, 2H, *J* = 8.4 Hz, ArH), 7.41–7.54 (m, 3H, ArH), 7.76–7.85 (m, 2H, ArH). HRMS calcd for C_22_H_22_ClN_3_ (M + H)^+^, 364.1575; found, 364.1577.

*(E)-4-(((Naphthalen-1-ylmethyl)imino)(piperazin-1-yl)methyl)benzonitrilehydrochloride (***II-8***)*: Yield 30%, Mp: 158–160 °C. ^1^H-NMR (400 MHz, D_2_O): δ 3.34 (br, 2H, piperazinyl-H), 3.63 (br,4H, piperazinyl-H), 4.18 (br, 2H, piperazinyl-H), 4.84 (s, 2H, -CH_2_-), 7.19 (d, 1H, *J* = 7.2 Hz, ArH), 7.40–7.63 (m, 6H, ArH), 7.77 (d, 2H, *J* = 8.4 Hz, ArH), 7.88 (d, 1H, *J* = 8.1 Hz, ArH), 7.94 (d, 1H, *J* = 9.0 Hz, ArH). HRMS calcd for C_23_H_22_N_4_ (M + H)^+^, 355.1917; found, 355.1921.

*(E)-3-(((Naphthalen-1-ylmethyl)imino)(piperazin-1-yl)methyl)benzonitrilehydrochloride (***II-9***)*: Yield 11%, Mp: 168–170 °C. ^1^H-NMR (400 MHz, D_2_O): δ 3.16 (br, 2H, piperazinyl-H), 3.45 (br,4H, piperazinyl-H), 4.04 (br, 2H, piperazinyl-H), 4.72 (d, 1H, *J* = 13.5 Hz, -CH′-), 4.78 (d, 1H, *J* = 13.5 Hz, -CH′′-), 6.85 (d, 1H, *J* = 5.7 Hz, ArH), 7.01–7.05 (m, 2H, ArH), 7.24–7.29 (m, 2H, ArH), 7.35–7.52 (m, 3H, ArH), 7.73–7.90 (m, 3H, ArH). HRMS calcd for C_23_H_22_N_4_ (M + H)^+^, 355.1917; found, 355.1911.

*(E)-1-(Naphthalen-1-yl)-N-(piperazin-1-yl(p-tolyl)methylene)methanaminehydrochloride (***II-10***)*: Yield 49%, Mp: 178–180 °C. ^1^H-NMR (400 MHz, D_2_O): δ 2.38 (s, 3H, -CH_3_), 3.19 (t, 2H, *J* = 5.4 Hz, piperazinyl-H), 3.42 (t, 2H, *J* = 5.7 Hz, piperazinyl-H), 3.51 (t, 2H, *J* = 5.4 Hz, piperazinyl-H), 3.96 (t, 2H, *J* = 5.4 Hz, piperazinyl-H), 4.74 (s, 2H, -CH_2_-), 7.10–7.15 (m, 3H, ArH), 7.21 (d, 2H, *J* = 8.1 Hz, ArH), 7.30–7.35 (m, 1H, ArH), 7.37–7.44 (m, 2H, ArH), 7.46–7.52 (m, 1H, ArH), 7.77 (d, 1H, *J* = 8.1 Hz, ArH), 7.83 (d, 1H, *J* = 9.3 Hz, ArH). HRMS calcd for C_23_H_25_N_3_ (M + H)^+^, 344.2121; found, 344.2108.

*(E)-N-((5-Nitronaphthalen-1-yl)(piperazin-1-yl)methylene)-1-phenylmethanaminehydrochloride(***III-1***)*: Yield 14%, Mp: 173–175 °C. ^1^H-NMR (400 MHz, D_2_O): δ 3.14–3.26 (m, 2H, piperazinyl-H), 3.51–-3.57 (m, 2H, piperazinyl-H), 3.71 (br, 2H, piperazinyl-H), 4.34 (br, 2H, piperazinyl-H), 4.23 (d, 1H, *J* = 15.0 Hz, -CH′-), 4.36 (d, 1H, *J* = 15.0 Hz, -CH′′-), 6.73 (d, 2H, *J* = 6.6 Hz, ArH), 7.06–7.16 (m, 3H, ArH), 7.59 (t, 1H, *J* = 7.8 Hz, ArH), 7.80–7.86 (m, 2H, ArH), 7.94 (t, 1H, *J*=7.2Hz, ArH), 7.29 (d, 1H, *J* = 7.8 Hz, ArH), 8.74 (d, 1H, *J* = 8.7 Hz, ArH). HRMS calcd for C_22_H_22_N_4_O_2_ (M + H)^+^, 375.1816; found, 375.1815.

### 3.2. Pharmacological Method

#### 3.2.1. Inhibition of 5-HT or NE Reuptake Activity in Rat Brain Synaptosome

All animal experiments were conducted in compliance with the Care and Use of Laboratory Animals, with the approval of Peking Union Medical College and Chinese Academy of Medical Sciences’ Animal Studies Committee (Project code, 7102116; October 2010). Adult male Wistar rats were used. Animals were killed by decapitation and the whole brain, with the exception of the brainstem and cerebellum, was quickly removed. The procerebrum region was prepared, weighed, and homogenized in 10 volumes of ice-cold 0.32 mol/L sucrose solution using a glass homogenizer. The homogenate was centrifuged at 1000× *g* and 4 °C for 10 min. The supernatant was decanted and used for the uptake experiments. In the assay, the tissue suspension (200 µL) was incubated with 50 nmol/L ^3^H-NE or 3H-5-hydroxytryptamine (800 µL) in Krebs-Henseleit bicarbonate buffer and a solution of the drug at the appropriate concentration (20 µL; 1 × 10^−4^, 1 × 10^−5^, 1 × 10^−6^, 1 × 10^−7^, 1 × 10^−8^ mol/L) or the vehicle (20 µL) at 37 °C for 10 min. For each assay, three tubes were incubated with the vehicle (20 µL) at 0 °C in an ice bath. After incubation, the tubes were immediately filtered with a cell harvester. The fiberglass filter membrane was washed three times with ice-cold saline, placed in a scintillation vial, and counted in liquid scintillation cocktail (4 mL). Active uptake was the difference between the counts per minute at 37 and 0 °C. The percent inhibition at each drug concentration was the mean of three measurements [15].

#### 3.2.2. TST in Rats

Rats were administered the vehicle as a negative control, duloxetine (30 mg/kg) as a positive control, or test compounds **I-19**, **II-4**, and II-5 (30 mg/kg). For two days before the formal experiment, the rats in each group were given doses orally once a day in the morning and in the afternoon. The rats were given doses orally once in each group in the formal experiment. After 60 min of administration, the rats were suspended head-down more than 50 cm from the ground with a 12-cm-long piece of insulating tape at a distance of 1 cm from the tail tip. The distance between adjacent rats was 15 cm. The immobility time of the rats was recorded. The rate of change in the immobility time of the rats in the test drug group and the positive control group was calculated and compared with the negative control group. Statistical analysis was performed using Prism 7.0 (GraphPad Software, San Diego, CA, USA). The data of the immobility time of vehicle, duloxetine, **I-19**, **II-4**, and **II-5** were analyzed by the One-way ANOVA.

#### 3.2.3. Acute Toxicity Test

Male ICR mice (20–25 g) were used from Institute of Materia Medica, Chinese Academy of Medical Sciences, Beijing, China. Mice were randomly divided into two groups with six mice each. Mice were orally given **II-5** with a single dose 200 and 400 mg/kg or vehicle control, respectively. The mouse death was monitored for 20 h after treatment. 

## 4. Conclusions

In this study, we designed and synthesized three series of arylamidine derivatives to discover potent reuptake inhibitors of 5-HT and NE transporters. Our results showed that series II compounds with a large aromatic ring exhibited improved in vitro 5-HT and NE inhibitory activity, and compound **II-5** was the most potent dual inhibitor (5-HT, IC_50_ = 620 nM; NE, IC_50_ = 10 nM). Compounds **I-19**, **II-4**, and **II-5** were selected for TST profiling in rats to test the in vivo antidepressant effect. These three compounds reduced the immobility time in the TST, indicating in vivo antidepressant activity. Compound **II-5** showed the most potent in vivo antidepressant activity and had an acceptable safety profile. These arylamidine derivatives are interesting compounds to explore further as potential antidepressant drug candidates.

## Figures and Tables

**Table 1:** Inhibition data for serotonin (5-HT) and norephinephrine (NE) reuptake for series I~III.

Compd.	R_1_	R_2_	5-HT ^a^ (IC_50_) ^b^	NE ^a^ (IC_50_) ^b^
Duloxetine	-	-	86% (640 nM)	97% (49 nM)
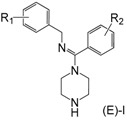				
**I-1**	H	4-OCH_3_	65%	67%
**I-2**	H	4-NO_2_	38%	66%
**I-3**	H	2-Cl	48%	65%
**I-4**	H	3-Cl	58%	63%
**I-5**	H	4-Cl	47%	57%
**I-6**	H	4-F	17%	39%
**I-7**	H	2-F	38%	35%
**I-8**	H	4-CN	54%	67%
**I-9**	H	3-CN	65%	51%
**I-10**	H	4-CH_3_	61%	47%
**I-11**	H	2-Cl, 4-NO_2_	50%	49%
**I-12**	4-Cl	4-NO_2_	51%	81%
**I-13**	4-Cl	2-Cl	67%	84%
**I-14**	4-Cl	3-Cl	76%	60%
**I-15**	4-Cl	4-Cl	48%	68%
**I-16**	4-Cl	4-F	45%	26%
**I-17**	4-Cl	2-F	43%	27%
**I-18**	4-Cl	4-CN	75%	63%
**I-19**	4-Cl	3-CN	82% (830 nM)	78% (560 nM)
**I-20**	4-Cl	4-CH_3_	72%	35%
**I-21**	4-OCH_3_	4-NO_2_	34%	57%
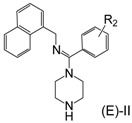				
**II-1**	-	H	80%	67%
**II-2**	-	4-NO_2_	57%	52%
**II-3**	-	4-F	84%	76%
**II-4**	-	2-F	90% (130 nM)	78% (880 nM)
**II-5**	-	2-Cl	81% (620 nM)	92% (10 nM)
**II-6**	-	3-Cl	65%	75%
**II-7**	-	4-Cl	62%	59%
**II-8**	-	4-CN	70%	46%
**II-9**	-	3-CN	88%	54%
**II-10**	-	4-CH_3_	82%	63%
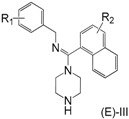				
**III-1**	H	5-NO_2_	73%	66%

^a^ Percent inhibition measured at a concentration of 10 μM, ^b^ IC_50_ was tested for selected compounds.

**Table 2:** The effect of four compounds in the rat test predictive of antidepressant activity.

Group	Dose (mg/kg)	Immobility Time (s)	Rate of Shorten Time (%)
Vehicle		54 ± 18	
Duloxetine	30	11 ± 5	79.0
**I-19**	30	34 ± 17	37.8
**II-4**	30	33 ± 19	39.7
**II-5**	30	18 ± 7	67.8

**Table 3:** Acute toxicity of **II-5** in mice.

Dose (mg/kg)	Mice (*n*)	Death (*n*)	Survival at 20 h
400	6	0	100%
200	6	0	100%
Vehicle	6	0	100%

## References

[1] Dilsaver S.C., Chen Y.W., Swann A.C., Shoaib A.M., Krajewski K.J. (1994). Suicidality in patients with pure and depressive mania. Am. J. Psychiatry.

[2] Schatzberg A.F. (2000). New indications for antidepressants. J. Clin. Psychiatry.

[3] Geneva. Depression Fact Sheet. http://www.who.int/mediacentre/factsheets/fs369/en/.

[4] Skolnick P., Popik P., Janowsky A., Beer B., Lippa A.S. (2003). “Broad spectrum” anti-depressants: Is more better for the treatment of depression?. Life Sci..

[5] Rakofsky J.J., Holtzheimer P.E., Nemeroff C.B. (2009). Emerging targets for antidepressanttherapies. Curr. Opin. Chem. Biol..

[6] Wróbel M.Z., Chodkowski A., Herold F., Gomółka A., Kleps J., Mazurek A.P., Pluciński F., Mazurek A., Nowak G., Siwek A. (2013). Synthesis and biologicalevaluation of novel pyrrolidine-2,5-dione derivatives as potential antide-pressant agents. Part 1. Eur. J. Med. Chem..

[7] Helguera A.M., Pérez-Garrido A., Gaspar A., Reis J., Cagide F., Vina D., Cordeiro M.N., Borges F. (2013). Combining QSAR classification models for predictive modeling of human monoamine oxidase inhibitors. Eur. J. Med. Chem..

[8] Goodwin G.M. (1996). How do antidepressants affect serotonin receptors? The role of serotonin receptors in the therapeutic and side effect profile of the SSRIs. J. Clin. Psychiatry.

[9] Subbaiah M.A.M. (2018). Triple Reuptake Inhibitors as Potential Therapeutics for Depressionand Other Disorders: Design Paradigm and DevelopmentalChallenges. J. Med. Chem..

[10] Shelton R.C. (2004). The dual-action hypothesis: Does pharmacology matter?. J. Clin. Psychiatry.

[11] Qin F., Guo Y.S., Wen H., Yang G.Z. (2009). Pharmacophore identification and comparison of serotonin/norepinephrine reuptake inhibitors. Acta Chim. Sin..

[12] Yang J., Wang X.F., Du G.H., Qin F., Wen H., Yang G.Z. (2007). Design, synthesis and activity evaluation of novel selective serotonin reuptake inhibitors. Chem. J. Chin. Univ..

[13] Yang J., Wang X.F., Wen H., Qin F., Yang G.Z. (2007). Design, synthesis and in vitro evaluation of phenylbenzamidine derivatives as SSRIs. Chin. Chem. Lett..

[14] Qin F., Yang J., Wen H., Zhang J.J., Wang Y.F., Ji C.X., Yang G.Z. (2009). Design, synthesis and 5-HT/NE dual reuptake inhibition activity of phenylbenzamidine derivatives. Chem. J. Chin. Univ..

[15] Vogel H.G., Vogel W.H., Schölkens B.A., Sandow J., Müller G., Vogel W.F. (2002). Drug Discovery and Evaluation—Pharmacological Assays.

